# Incident somatic comorbidity after psychosis: results from a retrospective cohort study based on Flemish general practice data

**DOI:** 10.1186/1471-2296-12-132

**Published:** 2011-11-29

**Authors:** Carla Truyers, Frank Buntinx, Jan De Lepeleire, Marc De Hert, Ruud Van Winkel, Bert Aertgeerts, Stefaan Bartholomeeusen, Emmanuel Lesaffre

**Affiliations:** 1 Department of General Practice, Katholieke Universiteit Leuven, Leuven, Belgium; 2 Research Institute Caphri, Maastricht University, Maastricht, The Netherlands; 3 University Psychiatric Center campus Kortenberg, Leuvensesteenweg 517, 3070 Kortenberg, Belgium; 4 L-Biostat, Katholieke Universiteit Leuven, Leuven, Belgium; Department of Biostatistics, Erasmus University Rotterdam, Rotterdam, The Netherlands

## Abstract

**Background:**

Psychotic conditions and especially schizophrenia, have been associated with increased morbidity and mortality. Many studies are performed in specialized settings with a strong focus on schizophrenia. Somatic comorbidity after psychosis is studied, using a general practice comorbidity registration network.

**Methods:**

Hazard ratios are presented resulting from frailty models to assess the risk of subsequent somatic disease after a diagnosis of psychosis compared to people without psychosis matched on practice, age and gender. Diseases studied are cancer, physical trauma, diabetes mellitus, gastrointestinal disorders, joint disorders, irritable bowel syndrome, general infections, metabolic disorders other than diabetes, hearing and vision problems, anemia, cardiovascular disease, alcohol abuse, lung disorders, mouth and teeth problems, sexually transmitted diseases.

**Results:**

Significant higher risks after a diagnosis of psychosis were found for the emergence of diabetes, physical trauma, gastrointestinal disorders, alcohol abuse, chronic lung disease and teeth and mouth problems. With regard to diabetes, by including the type of antipsychotic medication it is clear that the significant overall effect was largely due to the use of atypical antipsychotic medication. No significant higher risk was seen for cancer, joint conditions, irritable bowel syndrome, general infections, other metabolic conditions, hearing/vision problems, anaemia, cardiovascular disease or diabetes, in case no atypical antipsychotic medication was used.

**Conclusion:**

Significantly higher morbidity rates for some somatic conditions in patients with psychosis are apparent. People with a diagnosis of psychosis benefit from regular assessments for the emergence of somatic disorders and risk factors, including diabetes in case of atypical antipsychotic medication.

## Background

Psychotic conditions and especially schizophrenia have been associated with increased morbidity and mortality [1,2]. Overall mortality among patients with schizophrenia is reported to be about twice that in the general population [3]. Higher comorbidity can come from the illness itself and its consequences (e.g. lifestyle), medication use or neglect by the medical profession with regard to adequate screening and treatment for somatic comorbidity [4]. A number of reasons can account for this neglect, and the stigma related to psychiatric disorders is probably one of them [2].

We used data from Intego, a GP-based morbidity registration network in Flanders to study the incidence of subsequent somatic diseases in psychosis patients and to compare them to matched controls. Results can provide evidence to support or reject hypotheses found in literature. Based on literature, we expected increased incidences of general infections, physical trauma, metabolic conditions, gastro-intestinal and joint conditions, cardiovascular disease, alcohol abuse, chronic lung disease due to smoking, teeth- and mouth problems, and sexually transmitted diseases (STDs) [2,5,6].

## Methods

### Subjects and definitions

Data were obtained from Intego, a general practice-based morbidity registration network in Belgium, established at the Department of General Practice of the Katholieke Universiteit Leuven in 1994. The network provides data on the incidence and prevalence of all diseases presented to the GP either directly or through information provided by specialist consultation, in the case of psychosis most commonly a psychiatrist. Next to this, drug prescriptions and laboratory test results as well as some background information are recorded. GPs are selected to be included in the network on the basis of outstanding registration quality to maximize the validity and reliability of the data. By the end of 2007, the Intego database contained 86 GPs with over 2.1 million diagnoses in 197,000 different patients and covered more than 1.5% of the population in Flanders, the northern part of Belgium [7]. The population is representative for the Flemish population with regard to age, sex and socio-economic factors (SES). Data are recorded using a detailed internal thesaurus and subsequently classified according to ICPC-2 (International Classification of Primary Care), a classification system for morbidity in general practice accepted and used worldwide [8].

In this retrospective cohort study we considered the first episode of psychosis at all ages. Psychosis was defined as Schizophrenia, (ICPC-2 P72), Affective Psychosis (ICPC-2 P73), or Psychosis, NOS (ICPC-2 P98) taken together. All first diagnoses of psychosis from 1994-2007 coded in the above mentioned categories were used as the basis of the analysis (Table 1).

**Table 1:** Case definitions for ICPC-2 codes P72, P73 and P98

	ICPC-2		
ICD-10	Schizophrenia (P72)	Affective Psychosis (P73)	Psychosis, NOS (P98)
F20	Schizophrenia		
F21	Schizotypal disorder		
F22	Persistent delusional disorder		
F24	Induced delusional disorder		
F25	Schizoaffective disorder		
F28	Other nonorganic psychotic disorder		
F29	Unspecified nonorganic psychotic disorder		
F30		Manic episode	
F31		Bipolar affective disorder	
F34.0		Cyclothymia	
F23			Acute and transient psychotic disorder
F53.1			Severe mental and behavioural disorder associated with the puerperium

Cases were ascertained on the basis of the GP's decision: this could be his own diagnosis based on routinely collected data or based on a letter from the patient's psychiatrist. A patient was included as a case if he/she had at least one diagnosis in the categories mentioned above. Patients with organic psychosis or dementia were excluded from the analysis.

To assess the possible subsequent disease categories we combined diseases into meaningful outcome categories. These groups were also based on the ICPC-2 classification. Disease categories were defined on the basis of expert advice. Atypical antipsychotics were defined as ATC-class N05AH and N05AX.

### Statistical analysis

The risk of developing subsequent disease in patients with and without psychosis was analysed. All disease groups were analysed as possible consequences of psychosis. Patients who had a new diagnosis of a psychotic episode during the registration period (1 January 1994 to 31 December 2007), using the date of diagnosis as the baseline date, were included as cases. Non-psychosis patients were matched on age (with a range from plus and minus 5 years), gender, within a practice to create control subjects. The date of diagnosis of the index patient was assigned to the matched patients as a baseline date for the study. Therefore this date needed to fall within the first and last year a control was seen by the GP. Double use of control patients was not allowed. Patients with the particular disease before the psychosis date or matching date were excluded. We aimed for five controls to be selected for each case [9]. Both groups of patients were followed until they had an event or were censored at July 1st 2007.

For every disease the unadjusted hazard ratio for the association between psychosis and the disease was calculated (not controlled for age and gender).

Frailty proportional hazards analysis was used to identify the risk of a first episode of a somatic disease in patients with psychosis and matched controls without psychosis. Because of the association among the failure time data that might exist within different matches, these were modeled as random effect [10]. Random effect models have been used successfully in the analysis of correlated failure times. The approach assumed that there exists a common and unobserved latent variable, also called frailty, that characterizes the relationship or dependence of the correlated failure times [11]. The following simple frailty model was used:

$$h_{ij}\left(t\right)=h_{0}\left(t\right)\exp\left(\beta_{1}P_{ij}+\beta_{2}A_{ij}+\beta_{3}S_{ij}+v_{i}\right)$$

where *h*_0_(*t*) is the baseline hazard, *β*_1-3 _are the regression coefficients and *v_i _*is the unobserved random effect for every *i*-th match. Three covariates were used in the model: a group indicator P to denote whether a patient had experienced a psychosis or not, A for age at diagnosis for psychosis and S for the gender of the subject. To obtain consistent estimates, first the distribution was approximated by using piecewise baseline hazards based on estimated quantized intervals [12]. The model is then fitted using Gaussian quadrature in SAS PROC NLMIXED, using a normal distribution for the random effect [13].

For the analysis of diabetes, we stratified for the use of atypical versus other antipsychotic drugs [14].

To provide insight into a possible follow-up bias, data were calculated on the follow-up time of psychotic patients: are patients with a psychosis less inclined to visit their GP? Does this change after the diagnosis? We used the total years before and after the diagnosis (or matched date in case of the non-psychosis group) starting from 1994 and ending in 2008 and calculated in how many years patients were seen at least once during a year by the GP. For example if a patient was in the population in 1994 and still in 2008 and he/she had a psychosis diagnosis in 2003, the total years before was 9 and after 5. Let's assume this patient consulted the GP in 1999, 2000 and 2005 the ratio of consultation would be 0.22 before the diagnosis and 0.2 afterwards.

All statistical analyses were carried out using SAS, version 9.1.3 (SAS Institute, Cary, NC).

## Results

894 patients were diagnosed as suffering from a psychotic illness. The mean age at diagnosis was 48.8 years (SD = 21); 47 percent were males. Detailed epidemiological data are provided in another article [15]. The matched group included 4010 patients and the mean age was 45.5 (SD = 19) years. Gender distribution was the same as in the psychosis group.

Unadjusted (Table 2) and Adjusted (Table 3) hazard ratios for the development of the diagnostic categories in psychosis versus no psychosis subjects are shown. Data for STDs were not analysed further because of the small sample size (N = 42).

**Table 2:** Unadjusted Hazard ratios for the risk of subsequent disease after a diagnosis of psychosis

Is psychosis a risk factor for......?	Hazard ratio (95% CI) †
Cancer	0.85 (0.51-1.42)

**Physical trauma**	1.69 (1.29-2.2)****

**Diabetes (any antipsychotic medication)**	1.48 (0.92-2.38)

**Atypical antipsychotic Medication**	2.74 (1.41-5.33)**

Other antipsychotic Medication	1.03 (0.57-1.87)

**GI inflammation**	1.38 (1.01-1.89)*

Joint conditions	1.12 (0.86-1.45)

Irritable Bowel Syndrome	1.23 (0.25-6.10)

General infection	0.63 (0.34-1.16)

Metabolic conditions, no DM	0.97 (0.45-2.13)

Hearing/Vision	1.31 (0.75-2.29)

Anemia	0.68 (0.32-1.45)

Cardiovascular disease	0.93 (0.65-1.33)

**Alcohol abuse**	2.36 (1.14-4.86)*

**Chronic lung disease**	1.6 (1.17-2.38)**

**Teeth- and mouth problems**	1.58 (1.15-2.16)**

Sexually transmitted disease	4.48 (1.37-14.67)*

† Significant HRs are put in bold. *: p < 0.05; **: p < 0.01; ***: p < 0.001;****: p < 0.0001

**Table 3:** Hazard ratios for the risk of subsequent disease after a diagnosis of psychosis

Is psychosis a risk factor for......?	Hazard ratio (95% CI) †
Cancer	0.94 (0.56-1.58)

**Physical trauma**	1.72 (1.31-2.25)****

**Diabetes (any antipsychotic medication)**	1.77 (1.11-2.83)*

**Atypical antipsychotic Medication**	2.46 (1.29-4.71)**

Other antipsychotic Medication	0.94 (0.52-1.69)

**GI inflammation**	1.44 (1.04-1.98)*

Joint conditions	1.16 (0.88-1.52)

Irritable Bowel Syndrome	2.08 (0.57-7.59)

General infection	0.66 (0.36-1.22)

Metabolic conditions, no DM	1.11 (0.50-2.46)

Hearing/Vision	1.52 (0.87-2.65)

Anemia	0.89 (0.46-1.70)

Cardiovascular disease	1.02 (0.70-1.47)

**Alcohol abuse**	2.27 (1.10-4.69)*

**Chronic lung disease**	1.72 (1.20-2.47)**

**Teeth- and mouth problems**	1.63 (1.18-2.24)**

Sexually transmitted disease	-

† Significant HRs are put in bold. *: p < 0.05; **: p < 0.01; ***: p < 0.001;****: p < 0.0001

Significant higher risks after a diagnosis of psychosis (table 3 bold) were found for the emergence of physical trauma (HR = 1.72 (95% CI = [(1.31,2.25)]); GI inflammation (HR = 1.44 (95% CI = [1.04,1.98]); alcohol abuse (HR = 2.27 (95% CI = [1.10,4.69]); chronic lung disease (HR = 1.72 (95% CI = [1.20,2.47]) and teeth and mouth problems (HR = 1.63 (95% CI = [1.18,2.24]).

**Figure 1:**
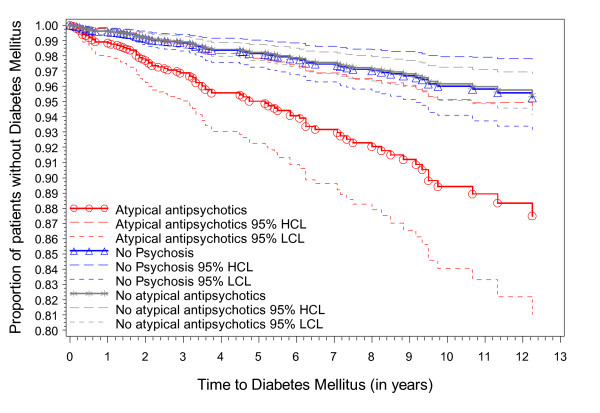
**Time to Diabetes Mellitus for different groups of antipsychotic treatment**.

More than 22% of these patients, diagnosed with psychotic illness, were treated with atypical antipsychotics, 45% with other antipsychotics, and 16% have received both. With regard to diabetes, by including the type of antipsychotic medication it was clear the significant overall effect (HR = 1.77 (95% CI = [(1.11,2.83)]) was largely due to the use of atypical antipsychotic medication (Figure 1). When this was entered in the model the effect of psychosis disappears and the effect of the atypical medication emerged (HR atypical medication = 2.46 (95% CI = [(1.29,4.71)]). Next to diabetes we also found a significant association of atypical medication and physical trauma (HR atypical medication = 1.82 (95% CI = [1.21, 2.75]). With regard to other antipsychotic medication an association was found with cardiovascular disease (HR = 1.6 (95% CI = [1.07,2.4]), alcohol abuse (HR = 4, 95% CI = [1.7, 9.4]) and mouth-and teeth problems (HR = 1.7, 95% CI = [1.16, 2.46]).

No significant higher risk was seen for cancer, joint conditions, IBS, general infections, other metabolic conditions, hearing/vision problems, anaemia and cardiovascular disease.

The follow-up time of patients showed consultation rates (95% CI) before diagnosis of psychosis (matched date in case of non-psychosis group) of 0.93 (0.92-0.94) for cases and 0.86 (0.85-0.87) for controls [16]. After diagnosis this was 0.89 (0.88-0.91) for cases and 0.84 (0.83-0.85) for matched controls.

## Discussion

Our study based on a large GP-based morbidity database indicates significantly higher morbidity rates for some somatic conditions in patients with psychosis: diabetes, physical trauma, GI inflammation, alcohol abuse, chronic lung disease and mouth and teeth problems, but surprisingly not for cardiovascular disease, although from literature this could be expected [17]. A similar study to ours however also found that older patients with psychotic disorders are diagnosed with cardiovascular diseases less frequently than other types of elderly patients. For younger patients there was no difference [16]. It could be hypothesized that the follow-up period is still too short for cardiovascular disease to emerge because a long period of exposure to risk factors is needed before cardiovascular disease emerges and/or that due to excess mortality in the psychosis group there is a selection bias. The relation requires further study. Many studies have found that patients with schizophrenia have increased rates of several chronic medical conditions, including coronary artery disease, chronic obstructive pulmonary disease, HIV, hepatitis C, and diabetes mellitus [18-20]. Goff et al. concluded that these are potentially reversible and preventable and that identifying and modifying risk factors could substantially improve the health of patients with schizophrenia [21]. Several hypotheses for this increased morbidity can be thought of. Patients with psychosis can be less inclined to take care of themselves. There is a high prevalence of smoking, obesity, poor diet, and sedentary lifestyle among patients with schizophrenia [22]. Adverse effects of antipsychotic medication may play a role [18], as was confirmed in this study and previous ones for atypical antipsychotics and subsequent diabetes [23,24]. It has been proposed that higher levels of psychiatric symptoms might lead to more somatic comorbidity because poor attention and poor insight might create an inability to self-monitor and follow medical regimens [19]. Careful follow-up might be a crucial factor with these patients.

Intego-data are representative for the Flemish population with regard to age, gender and SES. However to date no sufficient information is available on several lifestyle variables, such as smoking, which influences several chronic comorbid conditions [1,21]. We did not include data on weight or BMI, to investigate the relation between antipsychotic treatment, weight gain and diabetes. Because of the study design, the frequency of follow-up of weight, would not suffice to answer this relationship. Many of these events occur quite rapid after initiation of drug treatment and close monitoring is therefore needed [25]. To date, also no mortality data can be provided. Most other studies on somatic comorbidity in psychosis patients are cross-sectional, based in specialised settings, or using only younger schizophrenia patients. Because this study is a retrospective cohort study it can capture important longitudinal associations between psychosis and the development of somatic comorbidity over the whole population. We assume most diagnoses of psychosis in community dwelling people are known by the GP, either be it by their own diagnostic processes or via the diagnosis of a psychiatrist or hospital staff, which is transferred to the GP (however no quantitative data is available on this). A small number of institutionalized patients might 'disappear', however, in chronic psychiatric care without GP's involvement.

## Conclusion

Significantly higher morbidity rates for some somatic conditions in patients with psychosis are apparent. People with a diagnosis of psychosis benefit from regular assessments for the emergence of somatic disorders and risk factors, including diabetes in case of atypical antipsychotic medication. Primary care physicians should play an active role in ensuring that patients with mental illness are thoroughly followed. Because of the possible comorbidity shared care with other specialists such as psychiatrists, diabetologists, specialist nurses or other specialists should be established when necessary.

## Competing interests

The authors declare that they have no competing interests.

## Authors' contributions

CT was responsible for protocol development, statistical analysis and wrote the first draft of the paper. FB contributed to protocol development, study supervision, and data interpretation. JDL, MDH, RVW and BA contributed to the protocol design and data interpretation. SB contributed to data extraction and management and interpretation of data. EL contributed to protocol design, statistical analysis and interpretation of data. All authors read and approved the final manuscript.

## Pre-publication history

The pre-publication history for this paper can be accessed here:

http://www.biomedcentral.com/1471-2296/12/132/prepub
